# Bis(μ-5-chloro­quinolin-8-olato)-κ^3^ *N*,*O*:*O*;κ^3^ *O*:*N*,*O*-bis­[(acetato-κ^2^ *O*,*O*′)lead(II)]

**DOI:** 10.1107/S1600536809003559

**Published:** 2009-02-11

**Authors:** Gholamhossein Mohammadnezhad Sh., Mostafa M. Amini, Seik Weng Ng

**Affiliations:** a Department of Chemistry, General Campus, Shahid Beheshti University, Tehran 1983963113, Iran; b Department of Chemistry, University of Malaya, 50603 Kuala Lumpur, Malaysia

## Abstract

The mol­ecule of the title compound, [Pb_2_(C_9_H_5_ClNO)_2_(C_2_H_3_O_2_)_2_], lies about a center of inversion. The Pb^II^ atom is chelated by acetate and substituted quinolin-8-olate anions; the O atoms of the quinolin-8-olates also bridge to confer a five-coordinate status to each metal center. The geometry approximates a distorted Ψ-*fac* octa­hedron in which one of the sites is occupied by a stereochemically active lone pair.

## Related literature

The structural chemistry of lead(II) 8-hydroxy­quinolinates has been reviewed, including bis­(*μ*-acetato)diacetatotetra­kis(*μ*-quinolin-8-olato)tetra­lead dihydrate (Shahverdizadeh *et al.*, 2008 ▶).
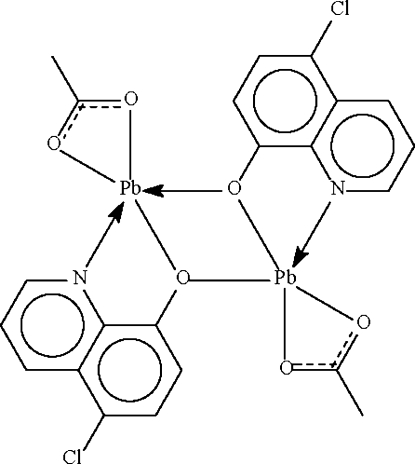


## Experimental

### 

#### Crystal data

[Pb_2_(C_9_H_5_ClNO)_2_(C_2_H_3_O_2_)_2_]
*M* *_r_* = 889.65
Monoclinic, 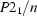

*a* = 5.3049 (1) Å
*b* = 11.8200 (3) Å
*c* = 17.4928 (3) Å
β = 94.569 (1)°
*V* = 1093.38 (4) Å^3^
*Z* = 2
Mo *K*α radiation
μ = 15.67 mm^−1^
*T* = 100 (2) K
0.10 × 0.03 × 0.02 mm

#### Data collection

Bruker SMART APEX diffractometer
Absorption correction: multi-scan (*SADABS*; Sheldrick, 1996 ▶) *T*_min_ = 0.303, *T*_max_ = 0.745
7713 measured reflections
1925 independent reflections
1655 reflections with *I* > 2σ(*I*)
*R*_int_ = 0.063

#### Refinement

*R*[*F* ^2^ > 2σ(*F* ^2^)] = 0.040
*wR*(*F* ^2^) = 0.107
*S* = 1.00
1925 reflections
155 parameters
72 restraints
H-atom parameters constrained
Δρ_max_ = 5.25 e Å^−3^
Δρ_min_ = −3.38 e Å^−3^

### 

Data collection: *APEX2* (Bruker, 2008 ▶); cell refinement: *SAINT* (Bruker, 2008 ▶); data reduction: *SAINT*; program(s) used to solve structure: *SHELXS97* (Sheldrick, 2008 ▶); program(s) used to refine structure: *SHELXL97* (Sheldrick, 2008 ▶); molecular graphics: *X-SEED* (Barbour, 2001 ▶); software used to prepare material for publication: *publCIF* (Westrip, 2009 ▶).

## Supplementary Material

Crystal structure: contains datablocks global, I. DOI: 10.1107/S1600536809003559/tk2365sup1.cif

Structure factors: contains datablocks I. DOI: 10.1107/S1600536809003559/tk2365Isup2.hkl

Additional supplementary materials:  crystallographic information; 3D view; checkCIF report

## Figures and Tables

**Fig. 1.**
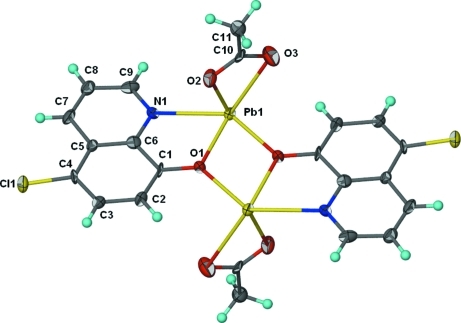
Thermal ellipsoid plot (Barbour, 2001) of Pb2(CH3CO2)2(C9H5ClNO)2; ellipsoids are drawn at the 70% probability level and H atoms of arbitrary radius. Unlabelled atoms are related by 1-x, 1-y, 1-z.

## supplementary crystallographic information

### Experimental

Lead acetate (0.38 g, 1 mmol) and 5-chloro-8-hydroxyquinoline (0.32 g, 2 mmol) were loaded into a convection tube; the tube was filled with dry methanol and kept at 333 K. Crystals were collected from the side arm after 1 day.

### Refinement

Carbon-bound H-atoms were placed in calculated positions (C—H 0.93–0.98 Å) and were included in the refinement in the riding model approximation, with *U*(H) set to 1.2–1.5*U*(C).

The crystal diffracted strongly owing to the presence of the heavy metal atom. However, this introduced severe absorption problems that could not be corrected analytically as the crystal did not have regular faces. Although a sphere of reflections was measured, multi-scan treatment only marginally improved the quality. The final difference Fourier map had large peaks/deep holes near the lead atom. The anisotropic displacement factors of the carbon atoms were restrained to be nearly isotropic.

### Crystal data

**Table:**

[Pb_2_(C_9_H_5_ClNO)_2_(C_2_H_3_O_2_)_2_]	*F*(000) = 816
*M**_r_* = 889.65	*D*_x_ = 2.702 Mg m^−^^3^
Monoclinic, *P*2_1_/*n*	Mo *K*α radiation, λ = 0.71073 Å
Hall symbol: -P 2yn	Cell parameters from 4123 reflections
*a* = 5.3049 (1) Å	θ = 2.9–28.3°
*b* = 11.8200 (3) Å	µ = 15.67 mm^−^^1^
*c* = 17.4928 (3) Å	*T* = 100 K
β = 94.569 (1)°	Yellow, prism
*V* = 1093.38 (4) Å^3^	0.10 × 0.03 × 0.02 mm
*Z* = 2	

### Data collection

**Table:**

Bruker SMART APEX diffractometer	1925 independent reflections
Radiation source: fine-focus sealed tube	1655 reflections with *I* > 2σ(*I*)
graphite	*R*_int_ = 0.063
ω scans	θ_max_ = 25.0°, θ_min_ = 2.1°
Absorption correction: multi-scan (*SADABS*; Sheldrick, 1996)	*h* = −6→6
*T*_min_ = 0.303, *T*_max_ = 0.745	*k* = −13→14
7713 measured reflections	*l* = −20→20

### Refinement

**Table:**

Refinement on *F*^2^	Primary atom site location: structure-invariant direct methods
Least-squares matrix: full	Secondary atom site location: difference Fourier map
*R*[*F*^2^ > 2σ(*F*^2^)] = 0.040	Hydrogen site location: inferred from neighbouring sites
*wR*(*F*^2^) = 0.107	H-atom parameters constrained
*S* = 1.00	*w* = 1/[σ^2^(*F*_o_^2^) + (0.0761*P*)^2^] where *P* = (*F*_o_^2^ + 2*F*_c_^2^)/3
1925 reflections	(Δ/σ)_max_ = 0.001
155 parameters	Δρ_max_ = 5.25 e Å^−^^3^
72 restraints	Δρ_min_ = −3.38 e Å^−^^3^

### Special details

**Table:**

Geometry. All e.s.d.'s (except the e.s.d. in the dihedral angle between two l.s. planes) are estimated using the full covariance matrix. The cell e.s.d.'s are taken into account individually in the estimation of e.s.d.'s in distances, angles and torsion angles; correlations between e.s.d.'s in cell parameters are only used when they are defined by crystal symmetry. An approximate (isotropic) treatment of cell e.s.d.'s is used for estimating e.s.d.'s involving l.s. planes.

### Fractional atomic coordinates and isotropic or equivalent isotropic displacement parameters (Å^2^)

**Table:**

	*x*	*y*	*z*	*U*_iso_*/*U*_eq_	
Pb1	0.63668 (6)	0.60161 (3)	0.421032 (17)	0.01511 (18)	
Cl1	−0.4232 (4)	0.30986 (19)	0.20373 (12)	0.0219 (5)	
O1	0.3463 (11)	0.4603 (5)	0.4439 (3)	0.0175 (13)	
O2	0.2902 (11)	0.7164 (6)	0.4314 (4)	0.0244 (14)	
O3	0.6097 (12)	0.8054 (6)	0.4933 (4)	0.0318 (16)	
N1	0.3482 (13)	0.5526 (6)	0.3014 (4)	0.0164 (15)	
C1	0.1685 (16)	0.4300 (8)	0.3908 (5)	0.0149 (17)	
C2	−0.0268 (17)	0.3547 (8)	0.4053 (5)	0.0186 (18)	
H2	−0.0351	0.3259	0.4557	0.022*	
C3	−0.2105 (16)	0.3205 (7)	0.3474 (5)	0.0186 (19)	
H3	−0.3392	0.2691	0.3594	0.022*	
C4	−0.2057 (16)	0.3610 (8)	0.2735 (5)	0.0154 (17)	
C5	−0.0195 (16)	0.4416 (7)	0.2573 (5)	0.0143 (18)	
C6	0.1645 (16)	0.4747 (7)	0.3134 (5)	0.0179 (18)	
C7	−0.0074 (17)	0.4913 (8)	0.1825 (5)	0.0203 (19)	
H7	−0.1270	0.4707	0.1416	0.024*	
C8	0.1763 (17)	0.5679 (8)	0.1710 (5)	0.0193 (19)	
H8	0.1863	0.6018	0.1221	0.023*	
C9	0.3506 (19)	0.5960 (7)	0.2321 (6)	0.021 (2)	
H9	0.4784	0.6497	0.2232	0.025*	
C10	0.3841 (15)	0.7984 (8)	0.4697 (5)	0.0172 (18)	
C11	0.202 (2)	0.8912 (7)	0.4903 (6)	0.025 (2)	
H11A	0.1228	0.8698	0.5369	0.037*	
H11B	0.0713	0.9013	0.4481	0.037*	
H11C	0.2953	0.9622	0.4992	0.037*	

### Atomic displacement parameters (Å^2^)

**Table:**

	*U*^11^	*U*^22^	*U*^33^	*U*^12^	*U*^13^	*U*^23^
Pb1	0.0176 (2)	0.0145 (2)	0.0126 (2)	−0.00021 (12)	−0.00237 (15)	0.00053 (12)
Cl1	0.0203 (10)	0.0240 (11)	0.0198 (11)	−0.0012 (9)	−0.0085 (9)	−0.0023 (9)
O1	0.021 (3)	0.014 (3)	0.017 (3)	−0.005 (2)	−0.005 (3)	0.001 (3)
O2	0.022 (3)	0.027 (4)	0.023 (3)	0.006 (3)	−0.007 (3)	−0.005 (3)
O3	0.026 (4)	0.027 (4)	0.041 (4)	0.000 (3)	−0.004 (3)	−0.012 (3)
N1	0.022 (4)	0.012 (4)	0.015 (4)	−0.003 (3)	−0.002 (3)	0.000 (3)
C1	0.017 (3)	0.017 (3)	0.010 (3)	0.002 (3)	−0.005 (3)	−0.002 (3)
C2	0.021 (4)	0.021 (4)	0.013 (4)	−0.001 (4)	−0.003 (4)	0.003 (4)
C3	0.019 (4)	0.016 (4)	0.020 (4)	−0.001 (3)	−0.004 (3)	0.004 (3)
C4	0.016 (3)	0.015 (3)	0.013 (3)	−0.001 (3)	−0.008 (3)	−0.006 (3)
C5	0.020 (4)	0.013 (4)	0.009 (4)	0.003 (3)	−0.002 (3)	−0.001 (3)
C6	0.018 (4)	0.014 (4)	0.021 (4)	0.003 (3)	0.000 (3)	−0.001 (3)
C7	0.026 (4)	0.021 (4)	0.013 (4)	0.007 (4)	−0.006 (3)	0.001 (4)
C8	0.024 (4)	0.021 (4)	0.012 (4)	0.001 (4)	0.000 (4)	0.002 (4)
C9	0.025 (5)	0.017 (4)	0.022 (5)	0.000 (3)	0.008 (4)	0.006 (4)
C10	0.018 (4)	0.023 (4)	0.010 (4)	−0.005 (4)	−0.005 (3)	0.010 (4)
C11	0.031 (5)	0.020 (5)	0.022 (5)	0.003 (4)	−0.003 (4)	−0.004 (4)

### Geometric parameters (Å, °)

**Table:**

Pb1—O2	2.303 (6)	C2—H2	0.9500
Pb1—O1	2.328 (6)	C3—C4	1.380 (13)
Pb1—O1^i^	2.469 (6)	C3—H3	0.9500
Pb1—N1	2.559 (7)	C4—C5	1.417 (13)
Pb1—O3	2.729 (7)	C5—C6	1.384 (13)
Pb1—C10	2.848 (9)	C5—C7	1.441 (12)
Cl1—C4	1.722 (9)	C7—C8	1.356 (13)
O1—C1	1.319 (11)	C7—H7	0.9500
O1—Pb1^i^	2.469 (6)	C8—C9	1.397 (15)
O2—C10	1.257 (11)	C8—H8	0.9500
O3—C10	1.237 (10)	C9—H9	0.9500
N1—C9	1.317 (12)	C10—C11	1.524 (13)
N1—C6	1.370 (11)	C11—H11A	0.9800
C1—C2	1.404 (13)	C11—H11B	0.9800
C1—C6	1.451 (13)	C11—H11C	0.9800
C2—C3	1.407 (13)		
			
O2—Pb1—O1	82.3 (2)	C2—C3—H3	119.6
O2—Pb1—O1^i^	93.9 (2)	C3—C4—C5	119.1 (8)
O1—Pb1—O1^i^	66.3 (2)	C3—C4—Cl1	118.8 (7)
O2—Pb1—N1	76.6 (2)	C5—C4—Cl1	122.2 (7)
O1—Pb1—N1	67.5 (2)	C6—C5—C4	120.7 (8)
O1^i^—Pb1—N1	133.6 (2)	C6—C5—C7	116.7 (8)
O2—Pb1—O3	51.1 (2)	C4—C5—C7	122.6 (8)
O1—Pb1—O3	119.6 (2)	N1—C6—C5	123.4 (8)
O1^i^—Pb1—O3	79.5 (2)	N1—C6—C1	115.5 (8)
N1—Pb1—O3	121.9 (2)	C5—C6—C1	121.0 (8)
O2—Pb1—C10	25.6 (2)	C8—C7—C5	119.4 (9)
O1—Pb1—C10	101.6 (2)	C8—C7—H7	120.3
O1^i^—Pb1—C10	86.5 (2)	C5—C7—H7	120.3
N1—Pb1—C10	99.5 (2)	C7—C8—C9	119.0 (8)
O3—Pb1—C10	25.5 (2)	C7—C8—H8	120.5
C1—O1—Pb1	121.3 (5)	C9—C8—H8	120.5
C1—O1—Pb1^i^	124.2 (5)	N1—C9—C8	123.9 (8)
Pb1—O1—Pb1^i^	113.7 (2)	N1—C9—H9	118.1
C10—O2—Pb1	102.2 (5)	C8—C9—H9	118.1
C10—O3—Pb1	82.6 (5)	O3—C10—O2	124.1 (9)
C9—N1—C6	117.6 (8)	O3—C10—C11	119.2 (8)
C9—N1—Pb1	128.0 (6)	O2—C10—C11	116.7 (8)
C6—N1—Pb1	114.4 (6)	O3—C10—Pb1	71.9 (5)
O1—C1—C2	122.8 (8)	O2—C10—Pb1	52.2 (4)
O1—C1—C6	120.9 (8)	C11—C10—Pb1	168.8 (6)
C2—C1—C6	116.3 (8)	C10—C11—H11A	109.5
C1—C2—C3	122.1 (8)	C10—C11—H11B	109.5
C1—C2—H2	119.0	H11A—C11—H11B	109.5
C3—C2—H2	119.0	C10—C11—H11C	109.5
C4—C3—C2	120.7 (8)	H11A—C11—H11C	109.5
C4—C3—H3	119.6	H11B—C11—H11C	109.5
			
O2—Pb1—O1—C1	73.2 (6)	Cl1—C4—C5—C6	−175.1 (6)
O1^i^—Pb1—O1—C1	170.9 (8)	C3—C4—C5—C7	−177.6 (8)
N1—Pb1—O1—C1	−5.5 (6)	Cl1—C4—C5—C7	3.6 (12)
O3—Pb1—O1—C1	109.5 (6)	C9—N1—C6—C5	−1.4 (12)
C10—Pb1—O1—C1	90.1 (6)	Pb1—N1—C6—C5	176.2 (6)
O2—Pb1—O1—Pb1^i^	−97.7 (3)	C9—N1—C6—C1	−179.6 (8)
O1^i^—Pb1—O1—Pb1^i^	0.0	Pb1—N1—C6—C1	−2.0 (9)
N1—Pb1—O1—Pb1^i^	−176.4 (3)	C4—C5—C6—N1	−179.6 (8)
O3—Pb1—O1—Pb1^i^	−61.4 (3)	C7—C5—C6—N1	1.7 (12)
C10—Pb1—O1—Pb1^i^	−80.8 (3)	C4—C5—C6—C1	−1.5 (12)
O1—Pb1—O2—C10	138.8 (5)	C7—C5—C6—C1	179.8 (8)
O1^i^—Pb1—O2—C10	73.4 (5)	O1—C1—C6—N1	−2.8 (12)
N1—Pb1—O2—C10	−152.6 (6)	C2—C1—C6—N1	176.6 (8)
O3—Pb1—O2—C10	0.3 (5)	O1—C1—C6—C5	179.0 (8)
O2—Pb1—O3—C10	−0.3 (5)	C2—C1—C6—C5	−1.6 (12)
O1—Pb1—O3—C10	−49.2 (6)	C6—C5—C7—C8	−1.2 (12)
O1^i^—Pb1—O3—C10	−104.0 (5)	C4—C5—C7—C8	−179.9 (8)
N1—Pb1—O3—C10	31.3 (6)	C5—C7—C8—C9	0.4 (13)
O2—Pb1—N1—C9	93.9 (7)	C6—N1—C9—C8	0.6 (13)
O1—Pb1—N1—C9	−179.0 (8)	Pb1—N1—C9—C8	−176.6 (6)
O1^i^—Pb1—N1—C9	176.4 (6)	C7—C8—C9—N1	−0.1 (14)
O3—Pb1—N1—C9	69.1 (8)	Pb1—O3—C10—O2	0.4 (8)
C10—Pb1—N1—C9	82.3 (7)	Pb1—O3—C10—C11	177.7 (7)
O2—Pb1—N1—C6	−83.4 (6)	Pb1—O2—C10—O3	−0.5 (10)
O1—Pb1—N1—C6	3.7 (5)	Pb1—O2—C10—C11	−177.9 (6)
O1^i^—Pb1—N1—C6	−0.9 (7)	O2—Pb1—C10—O3	179.5 (8)
O3—Pb1—N1—C6	−108.1 (6)	O1—Pb1—C10—O3	137.8 (5)
C10—Pb1—N1—C6	−95.0 (6)	O1^i^—Pb1—C10—O3	72.9 (5)
Pb1—O1—C1—C2	−172.5 (7)	N1—Pb1—C10—O3	−153.5 (5)
Pb1^i^—O1—C1—C2	−2.6 (11)	O1—Pb1—C10—O2	−41.8 (6)
Pb1—O1—C1—C6	6.8 (10)	O1^i^—Pb1—C10—O2	−106.7 (5)
Pb1^i^—O1—C1—C6	176.8 (6)	N1—Pb1—C10—O2	27.0 (6)
O1—C1—C2—C3	−178.1 (8)	O3—Pb1—C10—O2	−179.5 (8)
C6—C1—C2—C3	2.6 (13)	O2—Pb1—C10—C11	10 (3)
C1—C2—C3—C4	−0.4 (14)	O1—Pb1—C10—C11	−32 (3)
C2—C3—C4—C5	−2.8 (13)	O1^i^—Pb1—C10—C11	−97 (3)
C2—C3—C4—Cl1	176.0 (7)	N1—Pb1—C10—C11	37 (3)
C3—C4—C5—C6	3.7 (13)	O3—Pb1—C10—C11	−170 (3)

Symmetry codes: (i) −*x*+1, −*y*+1, −*z*+1.
